# Mutational landscape of canine B-cell lymphoma profiled at single nucleotide resolution by RNA-seq

**DOI:** 10.1371/journal.pone.0215154

**Published:** 2019-04-24

**Authors:** Diana Giannuzzi, Laura Marconato, Luciano Cascione, Stefano Comazzi, Ramy Elgendy, Sara Pegolo, Alessio Cecchinato, Francesco Bertoni, Luca Aresu, Serena Ferraresso

**Affiliations:** 1 Department of Comparative Biomedicine and Food Science, University of Padua, Legnaro, Padua, Italy; 2 Centro Oncologico Veterinario, Sasso Marconi, Bologna, Italy; 3 Università della Svizzera italiana (USI), Institute of Oncology Research (IOR), Bellinzona, Switzerland; 4 Swiss Institute of Bioinformatics (SIB), Lausanne, Switzerland; 5 Department of Veterinary Medicine, University of Milan, Milan, Italy; 6 Department of Immunology, Genetics and Pathology, Uppsala University, Uppsala, Sweden; 7 Department of Agronomy, Food, Natural Resources, Animals and Environment, University of Padua, Legnaro, Padua, Italy; 8 Department of Veterinary Science, University of Turin, Grugliasco, Turin, Italy; Leiden University Medical Centre, NETHERLANDS

## Abstract

The genomic landscape in human B-cell lymphoma has revealed several somatic mutations and potentially relevant germline alterations affecting therapy and prognosis. Also, mutations originally described as somatic aberrations have been shown to confer cancer predisposition when occurring in the germline. The relevance of mutations in canine B-cell lymphoma is scarcely known and gene expression profiling has shown similar molecular signatures among different B-cell histotypes, suggesting other biological mechanisms underlining differences. Here, we present a highly accurate approach to identify single nucleotide variants (SNVs) in RNA-seq data obtained from 62 completely staged canine B-cell lymphomas and 11 normal B-cells used as controls. A customized variant discovery pipeline was applied and SNVs were found in tumors and differentiated for histotype. A number of known and not previously identified SNVs were significantly associated to MAPK signaling pathway, negative regulation of apoptotic process and cell death, B-cell activation, NF-kB and JAK-STAT signaling. Interestingly, no significant genetic fingerprints were found separating diffuse large B-cell lymphoma from indolent lymphomas suggesting that differences of genetic landscape are not the pivotal causative factor of indolent behavior. We also detected several variants in expressed regions of canine B-cell lymphoma and identified SNVs having a direct impact on genes. Using this brand-new approach the consequence of a gene variant is directly associated to expression. Further investigations are in progress to deeply elucidate the mechanisms by which altered genes pathways may drive lymphomagenesis and a higher number of cases is also demanded to confirm this evidence.

## Introduction

Lymphoma is the most frequent hematopoietic cancer in dog and comprises a heterogeneous group of malignancies of varying severity [1]. According to the World Health Organization (WHO) [2], the most frequent B-cell lymphoma (BCL) is diffuse large B-cell lymphoma (DLBCL) by far having the highest incidence, followed by marginal zone lymphoma (MZL), Burkitt lymphoma and follicular lymphoma (FL) [3,4]. Under a clinical perspective, BCLs are also categorized in aggressive (DLBCL and Burkitt Lymphoma) and indolent (FL and MZL) lymphomas. However, both classifications reflect partially the biological behavior. Considering these limitations, recent investigations have described the molecular bases underlying canine BCL. Frantz et al. [5] performed the first gene expression profiling (GEP) study highlighting molecular similarities between DLBCL and MZL. Next, for DLBCL, molecular signatures involving specific signaling pathways resembling the human counterpart (i.e. NF-kB, PI3K-AKT and JAK-STAT signatures) were identified [6,7,8]. Analysis of copy number variations (CNVs) *via* array comparative genomic hybridization (aCGH) has revealed recurrent gains in chromosome 13 and chromosome 31 [8,9,10] which appeared correlated to treatment response and survival [10]. Regarding epigenetic deregulation, a recent genome wide-DNA methylation study revealed three distinct DLBCL subgroups with different outcome [11].

Besides GEP, DNA methylation profiling and CNV analysis, another layer in understanding cancer origin and evolution is represented by genetic polymorphisms. The use of brand-new Next Generation Sequencing (NGS) platforms and the implementation of bioinformatic programs have recently shown promising results. In turn, the cost of sequencing dropped down in recent years, providing a vast amount of whole genome and exome/transcriptome data [12]. The number of NGS studies is also increasing in veterinary medicine, but unfortunately, data analysis tools tailored for canine species still need to be tested or developed.

In the present study, single nucleotide variants (SNVs) were identified in a group of dogs affected by BCL using a variant calling pipeline applied to whole transcriptome sequencing (RNA-seq) data. Healthy dogs and dogs affected by various BCL histotypes were included to pursue different aims: first, to ascertain SNVs differentiating controls from BCL and to determine if this distinction is retained when stratifying according to histotype *per se*; second, to investigate whether specific SNVs differentiate aggressive from indolent BCL (iBCL); third, to explore the prognostic relevance of SNVs in DLBCL and fourth, to investigate a possible correlation between SNVs and gene expression to achieve an integrated overview of SNVs in canine DLBCL.

## Materials and methods

### Dogs and samples

All the clinical data of the dogs included are reported in S1 Table. Briefly, lymph nodes were obtained from 11 healthy dogs (controls) and 62 dogs affected by BCL and classified by histology and immunohistochemistry in DLBCLs (n = 50), FLs (n = 7) and MZLs (n = 5) according to WHO classification of canine lymphoma [2]. A routine diagnostic approach, including CD3, CD20 and PAX5 antibodies, was performed to phenotype all the lymphomas. Two pathologists with documented experience in canine lymphoma pathology reviewed the slides. All dogs with BCL underwent a complete staging work-up, consisting of history and physical examination, complete blood cell count with differential, serum biochemistry profile (including lactate dehydrogenase), thoracic radiographs, abdominal ultrasound, cytological evaluation of liver and spleen regardless of their sonographic appearance, flow cytometry on lymph node, peripheral blood and bone marrow, and surgical removal of a peripheral lymph node to obtain a histopathological diagnosis and material for the vaccine generation. The treatment protocol used for dogs treated with chemo-immunotherapy has been previously published [13]. Briefly, dogs were treated with l-asparaginase (week 1), vincristine (week 2, 3, 4, 13), cyclophosphamide (week 2, 13), doxorubicin (week 7, 16), lomustine (week 10, 19), and prednisone (week 1 through 20). If owners wished to treat their dogs with immunotherapy, dogs also received an intradermal injection of 0.5 ml of an autologous vaccine with hydroxylapatite and tumor-derived heat shock protein peptide complexes [13] on weeks 4–7, 12, 16, 20, and 24.

Time to progression (TTP) was measured as the interval between initiation of treatment and progressive disease. Dogs not experiencing progressive disease at the end of the study or lost to follow-up before progression were censored for TTP analysis. Lymphoma-specific survival (LSS) was measured as the interval between initiation of treatment and lymphoma-related death.

Approval for this study was granted by the Ministero dell’Istruzione, dell’Universita’ e della Ricerca (MIUR) Ethical Board (Number RBSI14EDX9).

### Sample processing

All 73 samples were processed in the same laboratory and following the same protocols, starting from RNA extraction to sequencing. Total RNA was extracted independently using the AllPrep DNA/RNA Mini Kit (Qiagen, Hilden, Germany) according to the manufacturer’s instructions. RNA concentration and integrity were measured with a NanoDrop ND-1000 spectrophotometer and assessed through the Bioanalyzer 2100 instrument (Agilent Technologies, Santa Clara, CA, USA). A total of 73 non-normalized libraries for RNA sequencing experiments were prepared by using SureSelect-Strand Specific RNA-Seq Library Preparation kit (Agilent Technologies) and a single end sequencing (50 SE) was carried out on an Illumina HiSeq2500 (Illumina Inc., San Diego, CA, USA). Controls and DLBCLs were already employed in a previous gene expression study [8] while FLs and MZLs were added in the present study. Raw Illumina reads were deposited in the NCBI Sequence Read Archive (SRA) repository under the accession numbers reported in S2 Table.

### SNP calling and genotyping

Raw sequences were assessed for quality using the reports generated by FASTQC (http://www.bioinformatics.babraham.ac.uk/projects/fastqc/). Subsequently, low-quality regions and adapters were trimmed using Trimmomatic 0.36 [14], (parameters: ‘SE–phred33 ILLUMINACLIP:adapter_fasta.fa:2:30:6 SLIDINGWINDOW:5:20 HEADCROP:3 MINLEN:40). Filtered reads were then mapped to the CanFam3.1 reference genome (Broad Institute, Cambridge, MA; released Sep. 2011; downloaded from Ensembl release 87) using STAR [15] in *two-pass* mode. Sorting, read groups assignment and duplicates marking were performed by means of PICARD toolkit (http://broadinstitute.github.io/picard/).

SNP discovery and genotyping across all samples was performed simultaneously using HaplotypeCaller [16] under standard parameters according to GATK v.3.7 [17] Best Practices (https://software.broadinstitute.org/gatk/best-practices/).

Four different datasets were then created, defining the comparisons to be performed in subsequent analyses: all BCL samples versus controls, DLBCL versus controls, iBCL (i.e. FL + MZL) versus controls and iBCL versus DLBCL. For each dataset, a first hard filtering step was applied (options—clusterSize 3—clusterWindowSize 35—filterExpression "FS > 30.0"—filterName FS—filterExpression "QD < 2.0"—filterName QD–genotypeFilterExpression “DP < 6”—genotypeFilterName LowDP—setFilteredToNocall) to filter out SNVs in clusters (more than 3 SNPs in a window of 35 bases), and variants with QualByDepth (QD) < 2.0 and FisherStrand (FS) > 30 accounting for variant quality and strand bias; in addition, loci with individual read depth (DP) below 6 were set to no-call.

**Fig 1:**
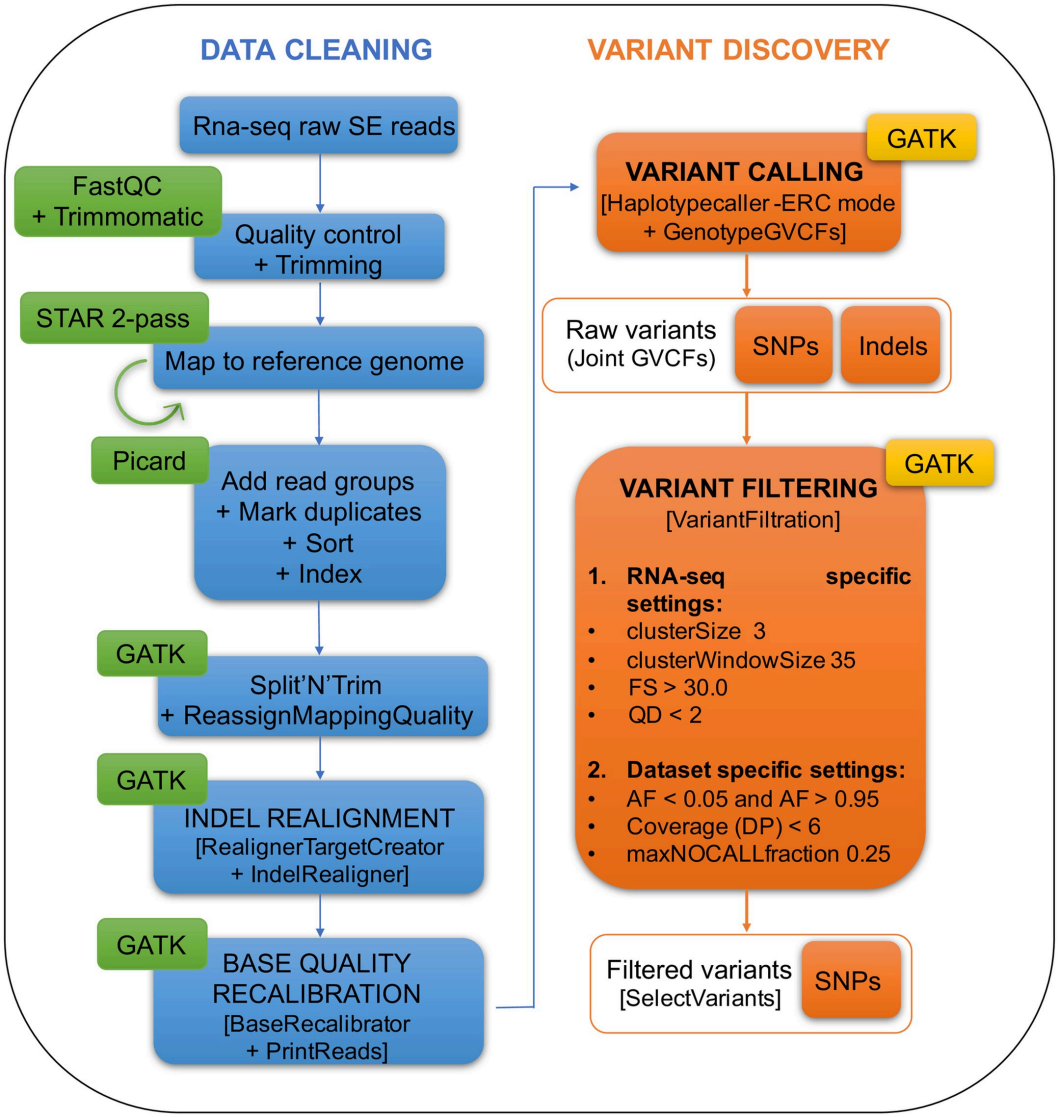
SNP calling analysis pipeline on RNA-seq data. This workflow was modified from GATK 3.7 Best Practices and customized according to our dataset.

A second filtering step (options—filterExpression "AF < 0.05"—filterName LowMAF—filterExpression "AF > 0.95"—filterName HighMAF) was applied to keep only SNVs with minor allele frequency (MAF) of at least 5%. At this level, only SNVs were considered and small insertions and deletions were excluded, the maximum allowed fraction of samples with no-call genotype was set to 0.25. The complete analysis workflow applied to each dataset is summarized in Fig 1.

After filtering, SNV were annotated with ANNOVAR [18] and Variant Effect Predictor (VEP) [19] to characterize the distribution of locations and consequences and to predict their putative functional effects.

To avoid calls related to known population variants, novel SNVs were identified filtering out previously published canine known population variation (dbSNP database version 146). Knowing that dbSNP contains also the “Lymphoma som SNPs” identified by Elvers et al. [20], the presence of those variants in our panels was manually checked separately.

### Case- control association analysis

VCF files were converted to PLINK format (.bed, .bim and .fam) using VCFtools [21].

For each previously defined dataset, a case-control association analysis was performed using a univariate linear mixed model (LMM) implemented in GEMMA v.0.96 software [22]. Only SNVs with MAF > 0.05 were included in the calculation of the kinship matrix. The Wald test was used to obtain p-values. To ensure sufficient correction for population stratification, inflation factors (λ) for each association were calculated in R software (https://cran.r-project.org). Benjamini-Hochberg (BH) adjusted p-values were determined with the function *p*.*adjust* for multiple test correction implemented in R. For each case-control association analysis, the significance threshold was set to a BH corrected p-value < 0.05.

PLINK software v.1.9 [23] was employed to calculate the principal components (PCs), to obtain unsupervised clustering and to achieve linkage disequilibrium analysis: default settings were used with commands—*pca*,—*cluster* and—*r2* respectively.

To test for significant differences in survival (TTP and LSS) across SNV clusters, Kaplan-Meier curves were generated and log-rank test was performed. Dogs treated with chemotherapy only (CH) and chemotherapy and immunotherapy (CH+IM) were tested as two separated groups. All the correlations between SNV clusters and clinicopathological features (i.e. age, sex, weight, stage and substage, bone marrow and blood infiltration, LDH blood levels and steroid pre-treatment) were tested with Fisher’s exact test.

### Functional analyses

To gain a functional annotation of genes harboring significant SNVs, Cytoscape’s plugin ClueGO [24] was used to identify relevant categories of biological processes, molecular functions, immune system processes and KEGG pathways. Being SNVs retrieved from RNA-seq data, only expressed genes were employed as reference background on ClueGO statistical analyses. The analysis was carried out on a total of 139 novel SNVs (DLBCL versus controls panel) corresponding to 129 genes (LMM association nominal p-value < 0.05), using right-sided hypergeometric tests and correction for multiple testing (FDR < 0.05). A relatively high kappa score (0.4) was used to increase the accuracy of estimation for the relationships between terms based on their overlapping genes.

The same 129 genes harboring significant novel SNVs resulting from case-control association were also crossed with differentially expressed genes (DEGs) (FDR < 0.01) analyzed on the same dataset [8].

A cluster comparison was then performed with ClueGO between 1,612 DEGs (FDR < 0.01 and logFC > 1.5 or logFC < -1.5) and the 1,561 genes harboring the 2,524 novel SNVs (DLBCL versus controls panel). To identify the most significant terms which were in common between the two lists, the following filtering criteria were applied: i) minimum % of genes associated with the term equal to 20; ii) minimum number of genes per term equal to 3; iii) FDR ≤ 0.01.

## Results

### Canine BCL genetic landscape

**Fig 2:**
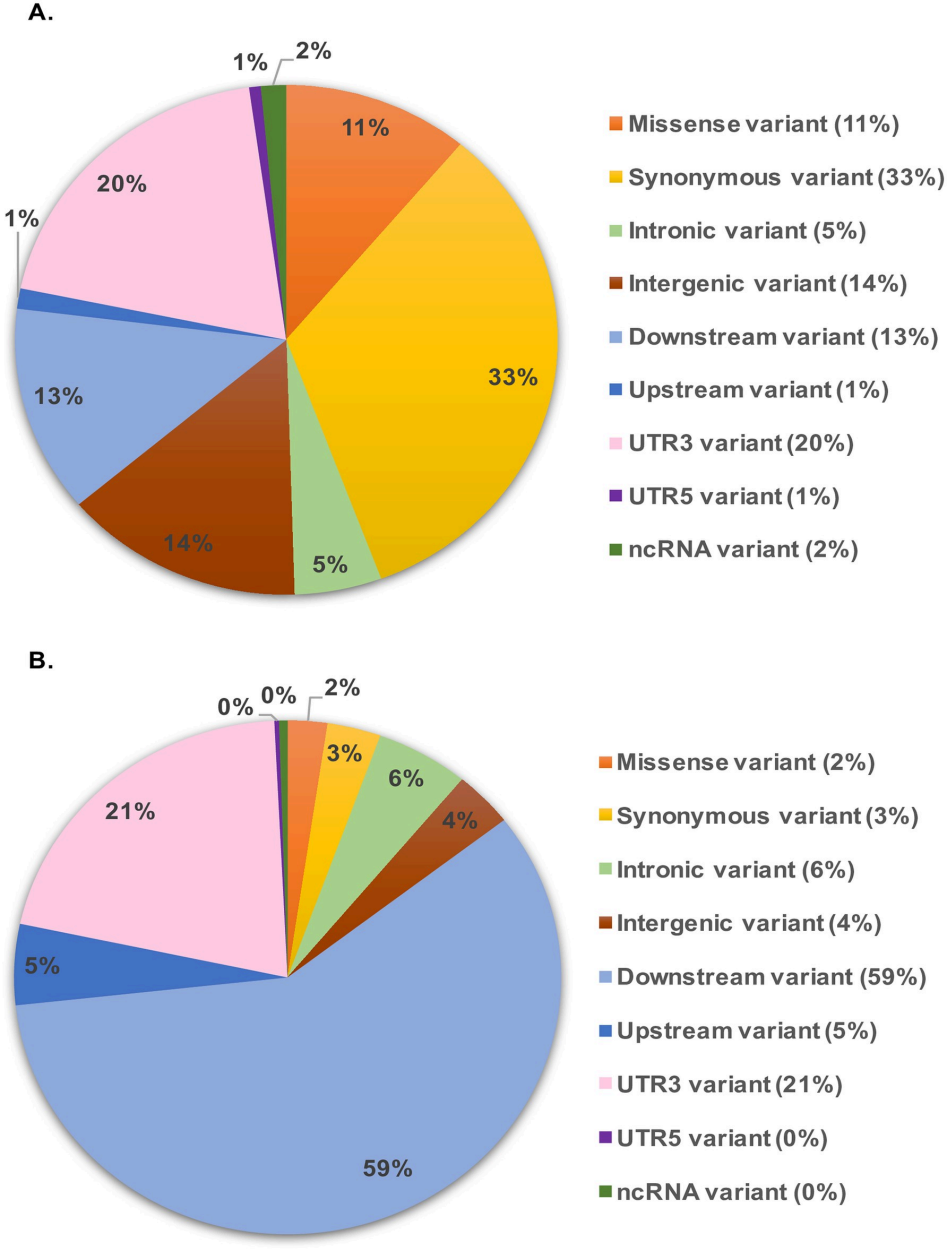
Pie charts depicting distribution and predicted consequences of SNVs in B-cell lymphomas versus controls panel. **A**. Distribution of all SNVs (n = 11,350). **B**. Distribution and consequences of novel SNVs (n = 2,598). Upstream/downstream variant: variant overlaps 1-kb region upstream/downstream of transcription start/end site, respectively.

RNA extracted from 62 canine BCL and normal B cells from 11 healthy dogs, used as controls underwent deep sequencing and an average of 33 million (M) reads per sample (range 24M-55M) was obtained. The mean percentage of reads uniquely mapped to the CanFam3.1 genome was 76.22%. Variant calling pipeline identified a total of 299,524 SNVs in at least one sample. After filtering steps, a total of 11,350, 11,235, 10,219 and 11,233 SNVs were retained in BCL versus controls, DLBCL versus controls, iBCL versus controls and iBCL versus DLBCL, respectively. After annotation with ANNOVAR, a total of 5,092 exonic SNVs were retrieved in BCL, 1,263 (25%) of which were classified as missense, stop-gain or stop-loss mutations (Fig 2). By using known variants from dbSNP, a total of 2,598 SNVs (22.9%) were classified as novel variants and 91 classified as missense, stop-gained or stop-loss, showing a predicted “moderate” to “high” impact.

When considering only DLBCL (DLBCL versus controls panel), a similar distribution was obtained with 2,524 SNVs (22.5%) assessed as novel variants, including 82 missense variants predicted having a “moderate” effect.

### Case-control association analysis reveals common signatures across BCL histotypes

**Fig 3:**
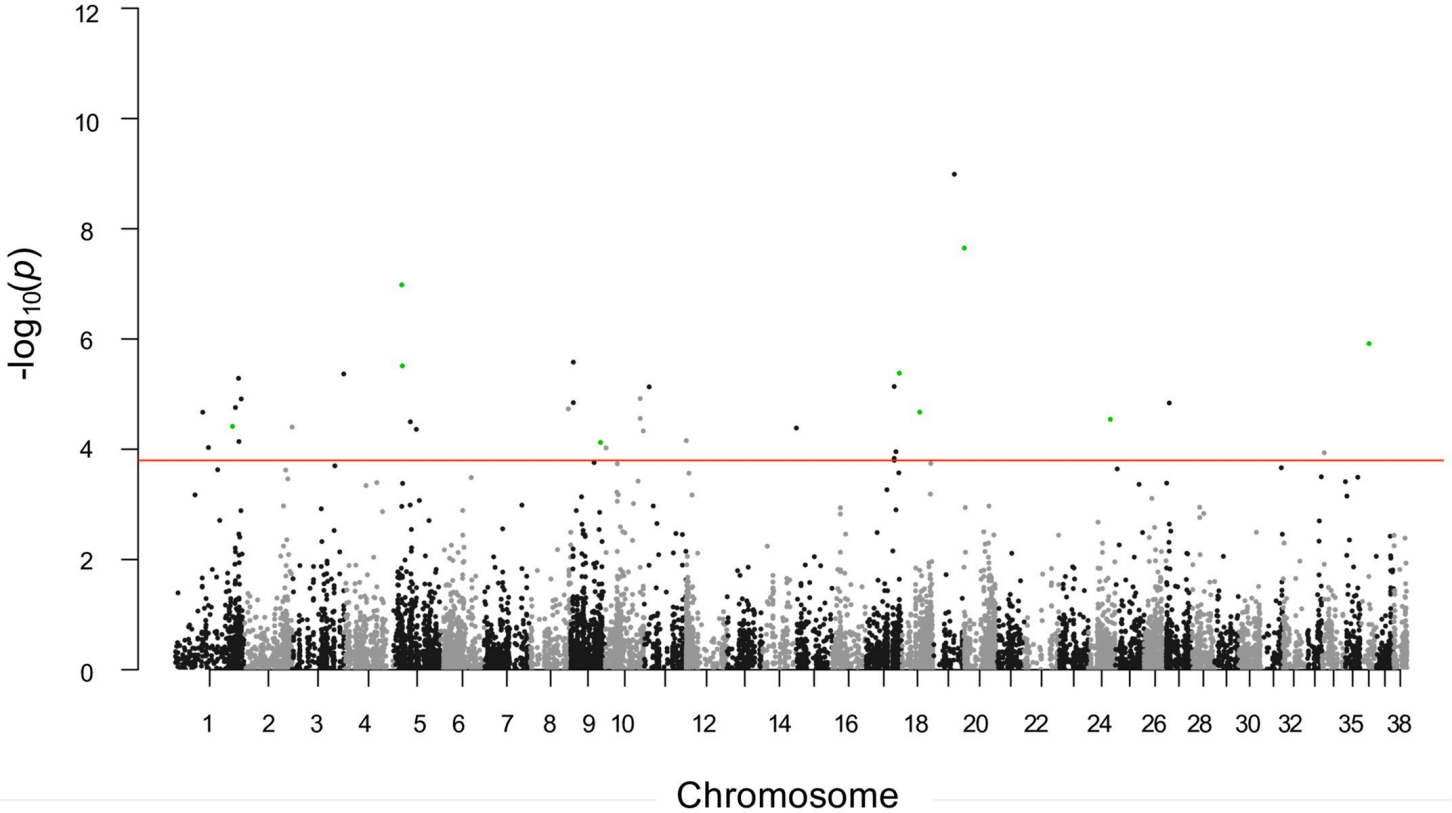
Manhattan plot of SNVs significantly associated with B-cell lymphomas. Manhattan plot of case-control association raw p-values on B-cell lymphomas. Horizontal red line: threshold of significance 0.05 (BH adjusted p-value); green dots: significant novel SNVs.

Using the BCL versus controls panel, case-control analysis revealed a total of 38 significant SNVs (λ = 1.06, BH adjusted p-value < 0.05, range = 0.048 to 2.44e-25) on *Canis familiaris* autosomes. After excluding known population variants retrieved from dbSNP, 11 novel SNVs were identified (Fig 3; S3 Table). Four were located in 3’ untranslated region (UTR 3’) of *UBE4A*, *SIDT2*, *CD44* and *FAM78A* genes, whereas five SNVs were annotated as exonic variants involving *RPN1*, *DYNC1I2*, *RFX5*, *ZNF787* and *KIAA0930*.

**Fig 4:**
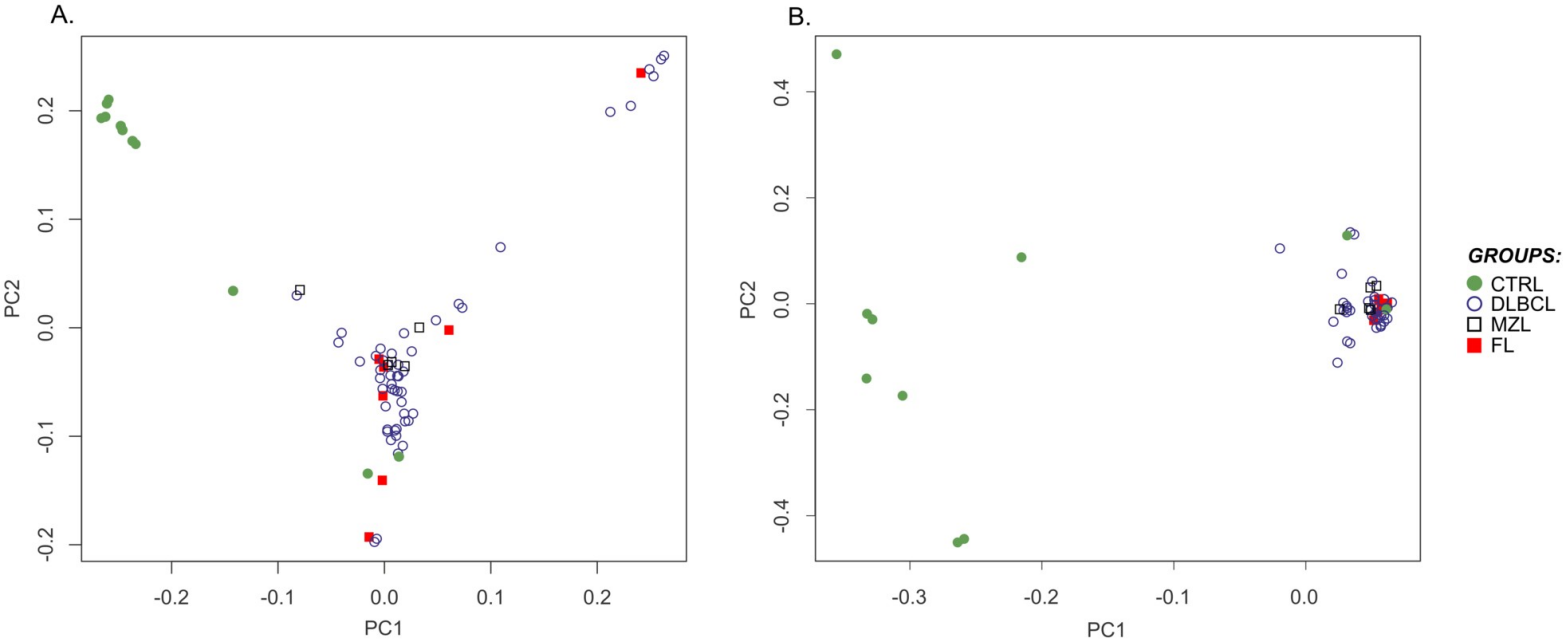
Principal components analysis on B-cell lymphomas versus controls panel. **A**. Samples distribution using all SNVs identified in the B-cell lymphomas (BCL) versus control panel. **B**. Samples distribution using only significant novel BCL-associated SNVs.

PCs analysis using all the SNVs (n = 11,350) divided samples into three clusters, one grouping controls and the other two composed by tumors. Notably, one BCL cluster included all the German shepherd dogs, highlighting the weight of the known population variants in samples distribution. Conversely, using only novel significant SNVs, all the BCLs clustered in a single group clearly well separated from controls (Fig 4).

Interestingly, a significant higher number of SNVs (n = 619; LMM association nominal p-value < 0.05) was located on chromosome 5 in BCL (Fisher’s exact test p-value 0.009). These loci showed a low inter-variant linkage disequilibrium (r^2^ < 0.2). However, when only novel SNVs (n = 148) were considered, statistical significance was not obtained.

The iBCL versus controls and the iBCL versus DLBCL case-control associations did not retrieve significant SNVs. However, in both panels the great majority were in common with the ones identified in BCL versus controls panel due to the limited number of indolent lymphoma cases. Only two significant SNVs detected in iBCL, named chr1:118062498 (T > C, nominal p-value 0.01) and chr8:72721506 (C > T, nominal p-value 0.01), carried relevant differences in allele frequencies compared to DLBCL.

Given the iBCL group limitations, all the subsequent analyses were focused on DLBCL, a morphologically homogeneous group accounting for the highest number of cases (n = 50). LMM association on DLBCL versus controls panel revealed 38 significant SNVs (λ = 1.08; BH adjusted p-value < 0.05, range = 0.047 to 6.34e-21). After filtering DLBCL significant SNVs for known population variants, a total of 9 novel SNVs were retained. Of them, 8 were located in exonic/UTR regions and two were missense variants (S3 Table).

To explore whether SNVs may influence clinical behavior, a specific only-DLBCL panel consisting of 11,672 SNVs was defined. After filtering known population variants, 2,525 novel SNVs were obtained. The latter were further filtered to keep only those previously found significant (nominal p-value < 0.05) in DLBCL versus controls LMM association analysis, obtaining a total of 69 significant novel SNVs.

These two sets of variants were employed separately by unsupervised clustering using k-means clustering (k = 3). Three clusters were generated from the two set of variants: DLBCL_1, DLBCL_2 and DLBCL_3 using all the novel variants belonging to the only-DLBCL panel while S_DLBCL_1, S_DLBCL_2 and S_DLBCL_3, using the 69 significantly DLBCL-associated novel SNVs. However, no clinicopathological features were significantly associated with the clusters (Fisher’s exact test p-value > 0.05). An exception was represented by the correlation of DLBCL_3 group with breed (Fisher’s exact test p-value 5.675e-05). Kaplan-Meier curves for TTP and LSS were also performed to explore the divergence in terms of survival among the clusters and, despite the log-rank test showing no significant differences, a trend was observed in the CH group of dogs, with DLBCL_1 and S_DLBCL_2 showing lower LSS and TTP compared to other clusters (S4 Table). Moreover, we explored whether clinicopathological features were significantly different in the two treatment groups, but none of them differentiated the two groups (Fisher’s exact test p-value > 0.05). Considering this inconclusive result, no further analyses were performed.

### SNVs functional annotation reveals overlaps with gene expression pathways

A list of significant DLBCL versus controls novel SNVs (n = 139; nominal p-value < 0.05) were assigned to 129 genes (from now defined as “Significantly Associated Genes”, SAGs). SAGs were then matched with DEGs identified in the same dataset by gene expression analysis [8] and 36 SAGs were found upregulated, whereas 10 down-regulated in DLBCL compared to controls.

Further, *LMNB1*, *ZC3H7A*, *MVB12A* and *ZNHIT6* were the only four genes harboring SNVs with predictable functional consequences. *LMNB1*, *ZC3H7A* and *MVB12A* concealed three missense variants (chr11:16038811:C > T, chr6:31068652:A > G, chr20:45367953:C > T, respectively) while *ZNHIT6* carried a 5’ untranslated region (UTR 5’) variant (chr6:62335585:G > C).

Again, using DLBCL versus controls panel, a SNP-based pathway analysis was conducted on SAGs in order to investigate biological processes significantly affected by genetic variations. To reach this aim, the list of 129 SAGs was employed. ClueGO analysis revealed 10 significantly enriched Gene Ontology (GO) biological process terms and 6 KEGG pathways (S5 Table). Pathways involved in apoptosis, immune system (*e*.*g*. TNF signaling pathway) and regulation of NF-kB signaling and MAPK cascade were the most highly impacted.

Finally, comparing the 1,561 genes harboring all the novel SNVs with the 1,612 DEGs, 74 enriched pathways resulted in common (S6 Table). Notably, negative regulation of programmed cell death, T-cell receptor signaling pathway, lymphocyte activation involved in immune response, B-cell activation, and JAK-STAT signaling pathway were included.

## Discussion

Improvement of NGS technologies has increased understanding of somatic mutations in both cancer origin and evolution, and knowledge about germline variations as risk factors for cancer development. A large amount of RNA-seq data were generated in the last years and, in addition to gene expression profiling, several bioinformatics pipelines were developed to render transcriptomic data suitable for variants identification [25,26]. Although the highest sensitivity and specificity in identifying genomic variants are ensured by merging genomic and transcriptomic data [25,27], the relative high coverage of RNA-seq enables to identify both germline and somatic SNVs in expressed genes at lower costs [25,26] and a recent study has also proved the high overlap and reliability of RNA-seq variant calling compared to whole exome sequencing (WES) data [28].

Mutational signatures distinguishing human DLBCL subgroups with different prognosis have been recently identified in several studies [29,30,31], whereas in dogs only one study explored somatic mutations in lymphoma using WES [20]. Despite the extreme relevance of the study, only three pure breeds were included; additionally, BCL were not stratified according to histotype and clinical data.

In the present study, we used pre-existing RNA-seq data derived from a gene expression study [8] and we applied a customized RNA-seq variant discovery pipeline for the canine species to identify novel SNVs in BCL. Since RNA-seq experimental design generally does not include matched tumor-normal samples, the individual genetic background increases the risk of considering population variants as tumor-associated mutations. In dog, this represents a considerable drawback due to the high breed-related genetic variability. Consequently, germline and somatic mutations are difficult to differentiate. To overcome this limit in our study, several stringent filtering steps were applied in order to exclude known population variations. This approach was only partially effective since novel SNVs PCs analysis showed a clear separation between BCL and controls. But, when only DLBCL-associated novel SNVs were considered, unsupervised clustering revealed a correlation between clusters and breeds, indicating a residual unfiltered bias due to the unavailability of individual genetic background information and incompleteness of database for canine population variations.

In order to find SNVs significantly associated with BCL, univariate LMM case-control association analysis was performed either grouping all the BCL together or separating DLBCL from iBCL into two independent datasets. Association analysis for BCL and DLBCL panels showed overlapping results with 89% of significant SNVs in common. This is explained by a higher prevalence of DLBCL in our study; however, a low genetic variability between DLBCL and iBCL is hypothesized.

To verify if iBCL hold peculiar genetic fingerprints, further analyses were performed comparing iBCL both with controls and DLBCL. Unfortunately, no SNVs reached the threshold for association significance in both comparisons. This was probably due to the higher heterogeneity within iBCL (grouping both MZL and FL) and to the limited number of samples. Further investigation on a bigger cohort is thus necessary to confirm this result. However, PCs analysis using all the SNVs did not separate iBCL from DLBCL, suggesting that mutational landscape might be not the pivotal causative factor for the indolent clinical behavior.

Supporting this hypothesis, a molecular similarity between MZL and DLBCL was already demonstrated using aCGH and GEP [5,7,9], suggesting that the two conditions might constitute a continuum of the same disease [32,33]. Furthermore, CNVs in FL are also similar to other BCL histotypes [34]. To investigate potential overlap with WES lymphoma-associated SNVs by Elvers et al. [20], both BCL versus controls and DLBCL versus controls panels were compared with “Lymphoma som SNPs” (only PASS-flagged) panel and only 4 SNVs resulted in common. The different sequencing target of WES and RNA-seq might explain this inconsistency. The association analysis using BCL panel identified a total of 38 significant SNVs (11 novel) of which 33 (10 novel) SNVs were located in known genes. Conversely, no genes were shared when comparing to Elvers et al. BCL-associated genes list, but it’s also true that only 38 genes from this WES list were expressed in our RNA-seq data, thereby preventing from assessing any conclusion. Similar results were obtained using all “Lymphoma som SNPs” list, where only 3% were located on expressed genes in our dataset. In addition, the low number of Cocker Spaniel and Golden Retriever in our dataset (15.6%) impeded a proper comparison.

Exploring gene function harboring SNVs with predictable impacting consequences, some valuable findings were retrieved. *LMNB1* encodes for a component of nuclear envelope involved in epigenetic chromatin regulation and it’s down-expressed in the majority of human germinal centre-derived lymphomas [35]. Particularly attractive is the missense variant on *RFX5*. This gene encodes one of the three subunits of the RFX protein that is a specific activator of major histocompatibility class II (MHCII) genes. Loss of MHCII antigens *via* chromosomal deletion is a known event in human DLBCL involving immune-privileged (IP) sites such as testes and brain [36] and often correlated to Epstein-Barr virus [37]. Conversely, in human non-IP DLBCL, MHCII loss of function is more often associated with an altered transcription event [38]. In canine BCL low MHCII protein expression is associated with poor outcome [39], but molecular basis beyond this event has never been investigated.

Notably, a significant higher number of SNVs was identified on chromosome 5 (chr5). Similarly, a recent genome-wide association study in Golden Retrievers demonstrated the presence of two predisposing loci on chr5 shared both in BCL and hemangiosarcoma [40].

The comparative analysis between SAGs and DEGs revealed a significant overlap of 74 pathways including negative regulation of programmed cell death, B-cell activation, JAK-STAT signaling cascade, lymphocyte differentiation and immune response. Most of them are also recurrently mutated in human DLBCL [41,42,43]. Interestingly, pathways involved in T cell differentiation and activation were also retrieved. In dogs, few studies on T regulatory cells were conducted to assess the prognostic impact and the modifications of T cell subsets in BCL surrounding microenvironment [44,45]. Emerging evidence in human DLBCL suggests that the impaired T-cell subpopulation distribution and the altered expression of their regulatory genes play a key role in tumorigenesis and cancer immune escape, and furthermore have a prognostic relevance [46,47]. Despite the lack of significance, in the CH group a lower median LSS was found in DLBCL_1 and S_DLBCL_2 compared to the other clusters. These two clusters showed no correlation with breeds, suggesting a reliable subgroup differentiation based on novel SNVs. Differently, SNV clusters in the CH+IM group didn’t show any association survival suggesting that immunotherapy treatment might act on pathways not strictly related to single nucleotide alterations. Further investigations are needed to address whether specific sets of SNVs hold the potential to discriminate DLBCL subgroups with prognostic relevance.

In conclusion, the present study provides the first description of RNA-seq-based SNV detection and genotyping in canine lymphoma. Genetic origins and molecular diversity of BCL were retrieved in different dog breeds and histotypes identifying SNVs associated to the disease. The innovative approach of the analysis conducted here opens new perspectives in veterinary medicine and mutations identified represent an initial dataset for investigation of genomic landscape of BCL in future. Thus, a more comprehensive approach using whole genome/exome sequencing approach will confirm the relevance of the detected SNVs. Considering cancer heterogeneity, mutational processes and breed differences [48], a larger cohort of samples to detect distinctive mutations is needed.

## Supporting information

S1 Table Clinical data of dogs included in the study. (XLSX)

S2 Table SRA repository accession numbers for RNA-seq data employed in the study. (XLSX)

S3 Table List of significantly associated SNVs from case-control association. S3 Table contains two worksheets organized as follow: S3.1 Significantly associated SNVs on BCL versus controls; S3.2 Significantly associated SNVs on DLBCL versus controls. Columns A-C: Dog variant ID and its position on CanFam3.1 genome. Column D: Minor allele. Column E: Major allele. Column F: allele frequency. Column G: beta estimates. Column H: standard errors for beta. Column I: remle estimates for lambda. Columns J, K: nominal p-value from Wald test and BH adjusted p-value. Columns L, M: Dog ENSEMBL gene ID and gene symbol, Column N: ANNOVAR and VEP variant annotation, Column O: dbSNP variant ID.
(XLSX)

S4 Table Median TTP and LSS for clusters obtained by unsupervised clustering using all (1, 2) and only significant (3, 4) novel SNVs from DLBCL. Dogs treated with chemotherapy-only and chemotherapy and immunotherapy were tested separately.
(XLSX)

S5 Table Significantly enriched pathways and gene ontology (GO) terms identified by ClueGO on DLBCL significantly associated genes (SAGs). Columns A, B: GO/KEGG terms ID and description. Column C, D: Number and percentage of overlapping genes. Column E: BH-adjusted (FDR) p-value. Column F: List of SAGs belonging to each GO/KEGG term.
(XLSX)

S6 Table Significantly enriched pathways and gene ontology (GO) terms in common between significantly associated genes (SAGs) and differentially expressed genes (DEGs) identified by ClueGO on DLBCL. Columns A, B: GO/KEGG terms ID and description. Column C, D: Number and percentage of genes in pathways that are either significantly mutated or differentially expressed. Column E: BH-adjusted (FDR) p-value. Column F, E: List and percentage of SAGs belonging to each GO/KEGG term. Column H, I: List and percentage of DEGs belonging to each GO/KEGG term.
(XLSX)
